# Identification and characterization of H-2^d^ restricted CD4^+^ T cell epitopes on Lpp20 of *Helicobacter pylori*

**DOI:** 10.1186/1471-2172-13-68

**Published:** 2012-12-12

**Authors:** Yan Li, Yin Jiang, Yue Xi, Lili Zhang, Jun Luo, Diandian He, Shuang Zeng, Yunshan Ning

**Affiliations:** 1 Institute of Biotherapy, School of Biotechnology, Southern Medical University, North1838 Guangzhou Road, Guangzhou 510515, PR China; 2 School of Nursing, Southern Medical University, North1838 Guangzhou Road, Guangzhou, 510515, PR China; 3 Department of Medical Microbiology, School of Public Health and Tropical Disease, Southern Medical University, North1838 Guangzhou Road, Guangzhou, 510515, PR China

**Keywords:** *Helicobacter pylori*, Lpp20, CD4^+^ T cell, Epitope

## Abstract

**Background:**

Previous investigation has demonstrated that CD4^+^ T cells play a crucial role in effective immunity against *Helicobacter pylori (H.pylori)* infection. It has been well proved that Lpp20 is one of major protective antigens that induce immune responses after *H.pylori* invades host. Therefore it is valuable to identify CD4^+^ T cell epitopes on Lpp20, which is uncharacterized.

**Methods:**

Putative epitopes of H-2^d^ restricted CD4^+^ T cell on Lpp20 of *H.pylori* were predicted by the SYFPEITHI algorithm and then eight hypothetical epitope peptides were synthesized**.** After BALB/c mice were primed with recombinant Lpp20, splenic CD4^+^ T cells were isolated and stimulated with synthesized peptides to measure T cell proliferation and MHC restriction. Cytokine profile was determined by ELISA and real-time PCR. Two identified epitopes were used to immunize mice to investigate CD4^+^ T cell response by flow cytometry.

**Results:**

Two of eight peptides were able to stimulate CD4^+^ T cell proliferation and were mapped to residues 83-97aa and 58-72aa on Lpp20 respectively. These two peptides additively stimulated Th1 cells to secrete IFN-γ. The percentage of CD4^+^ T cell from mice immunized with two identified epitopes respectively was higher than the control group.

**Conclusion:**

The identification and characterization of two CD4^+^ T cell epitopes of Lpp20 helps understand the protective immunity of Lpp20 in *H.pylori* infection and design effective epitope vaccines against *H.pylori*.

## Background

Helicobacter pylori(*H.pylori*) is a gram-negative bacilli, which colonizes the stomach and causes 50% infection of the population worldwide. Chronic infection with *H.pylori* is the main cause of various gastroduodenal diseases such as gastritis, peptic ulcer and is also a risk factor for gastric adenocarcinoma and lymphoma. In general, the host elicits robust immune response to *H.pylori* infection. However, the infection is often persistent and lasts for very long time, suggesting that *H.pylori* may evade both innate and adaptive immune responses.

The mechanism of protection against *H.pylori* infection remains largely unknown, but there are growing evidences to support a pivotal role of CD4^+^ T helper cells (Th cells) in the immune response against *H.pyori* infection [1]. Th cells are activated when the peptide antigens are presented by MHC II molecules, which are expressed on the surface of antigen presenting cells (APCs). Once activated, Th cells are divided rapidly and secrete cytokines to regulate the active immune response. When MHC II mutant mice are challenged by *H.pylori*, greater colonization of *H.pylori* in mutant mice are formed than in WT mice, suggesting that the mutation in MHC II on APC cells fails on the presentation of epitopes peptides. The level of anti *H.pylori* antibody in challenged wild-type mice is high but not detectable in mutant mice. Moreover, oral immunization with *H.pylori* whole-cell lysates reduced infection in wild-type and MHC I ^-/-^ mice but not in MHC II ^-/-^ mice [1]. All these observations suggest that Th cells may play an important role in protection of *H.pylori* infection.

Antibiotic treatment is the most effective therapy for *H.pylori* infection. Unfortunately, this strategy often results in many side-effects, such as poor patient compliance, production of antibiotic resistant strain and re-infection. Recently, developing effective vaccines against *H.pylori* has attracted much attention. Epitope-based vaccine design (EBVD) represents an alternative way to improve native antigen which can not evoke optimal immune response to pathogen [2]. The conserved epitopes identified by EBVD have been shown to not only elicit specific immune response but also increase potency and breadth of immune response. One of the key steps in EBVD approach is the identification of appropriate epitopes to obtain effective response.

Lpp20 is an outer membrane lipoprotein of *H.pylori* and also sheds from surface to medium [3,4]. Lpp20 is one of major antigens recognized by rabbit antiserum against *H.pylori*[5]. Passive anti-Lpp20 antibody transfusion decreases *H.pylori* infection in *H. pylori* infected mice [3]. Therefore, Lpp20 is an excellent vaccine candidate antigen for *H.pylori* infection.

In this study, we hypothesized that modified candidate antigens of Lpp20 may trigger more effective immune response induced by *H.pylori* infection. Two identified peptides on Lpp20 were able to stimulate CD4^+^ T cell proliferation and additive secretion of IFN-γ. These results provide us better understanding of cellular immunity against *H.pylori* infection and insights into the design of *H.pylori* vaccines.

## Methods

### Prediction and synthesis of Lpp20 Th cell epitopes

Amino acid sequence of Lpp20 for *H.pylori* strain NCTC11639 was acquired from NCBI protein database (No. AAZ13599 submitted by us [6]). For BALB/c mice, MHC II molecule includes I-A^d^ and I-E^d^ types. Potential I-A^d^ and I-E^d^ restricted Th cell epitopes of Lpp20 were predicted by SYFPEITHI system [7]. Five highest-scored I-A^d^ restricted and five highest-scored I-E^d^ restricted putative epitopes were selected. After further analysis, eight epitopes were synthesized by Chinese peptide Ltd.Co. (Hangzhou, China). The peptides were supplied as lysophilized powder and dissolved in dimethyl sulfoxide (DMSO) at a concentration of 4 mg/ml, sterilized with filter and stored in aliquots at −20°C. Peptides were diluted with RMPI 1640 incomplete culture medium (Gibco) before experiments. Recombinant Lpp20 (rLpp20) was expressed in Escherichia coli previously [6].

### Mice and immunization

Female BALB/c mice aged 6 ~ 8 weeks were purchased from Experimental Animal Center of Southern Medical University and approval to conduct this study was granted by the Ethics Committee of Southern Medical University. For rLpp20 immunization, mice were administered with 100 ug of rLpp20 emulsified in Complete Freund’s adjuvant (CFA; Sigma) by subcutaneous route in the four limbs and boosted 2 weeks later with the same protein in incomplete Freund’s adjuvant (IFA; Sigma). For peptides immunization, mice were administered with 100ug of peptide in IFA using the same procedure. Mice were used for experiments at 7–10 days after immunization. Mice immunized with PBS were used as control. Five mice were included in each group.

### Preparation of antigen presenting cell (APC)

Mice were sacrificed and spleens were harvested. After treatment with erythrocyte lysing buffer (0.83% NH_4_Cl_2_), splenocytes were resuspended to 5 × 10^7^ cells /ml in PBS with the addition of 100 μl mitomycin-C (Sigma; 500 μg/ml in PBS). Then these cells were incubated for 20 min at 37°C followed by three washes with RPMI 1640. The supernatant was discarded and the pelleted cells were resuspended in RPMI 1640 as APCs.

### CD4^+^ T cells proliferation assay

Mice were euthanized and spleens were harvested after the last immunization. Single-cell suspensions were obtained by homogenizing spleens and passing cells through a 70 mm cell strainer. The erythrocytes were removed by Ammonium Chloride Lysing Reagent (BD Biosciences, PharMingen). CD4^+^T cells were negatively sorted using mouse CD4 negative isolation kit (Dynal). Routinely, the resultant cells were >95% CD4^+^ T cell as determined by flow cytometry. To stimulate CD4^+^ T cells, peptides or rLpp20 were added into the mixture of APCs (4 × 10^5^) and CD4^+^ T cells (2 × 10^5^) which were resuspended in 200 μl RPMI-1640 complete cultured (RPMI-1640, 10% fetal bovine serum, 2 mM glutamine, 100 U of penicillin/ml, 100 U of streptomycin/ml, 50 mM 2-mercaptoethanol, and 25 mM HEPES). Culture without any antigen was served as negative control. Other controls were APCs without CD4^+^ T cells in the presence of ConA and CD4^+^ T cells without APC in the presence of peptides. Culture was set up in triplicate and incubated at 37°C, 5% CO_2_ for 5 days. During the final 16–18 h, each well was pulsed with 1 μCi of ^3^H] thymidine (Amersham Biosciences, Piscataway, NJ). ^3^H] thymidine incorporation was measured in a liquid scintillation counter after collecting cells onto glass fiber filters. The stimulation index (SI) was determined by comparing ^3^H] thymidine incorporation in the peptide-stimulated wells with unstimulated wells using the following equation: SI = mean cpm of peptide wells/mean cpm of no peptide wells. Experiments were independently repeated three times. Proliferative response was considered positive when SI was ≥2 at the 95% confidence level [8,9]. To investigate if there was any additive or subtractive interaction among the identified epitopes, the peptides were pooled to stimulate CD4^+^ T cells from mice immunized with rLpp20.

### MHC restriction studies

To detect MHC restriction for presentation, mAbs (eBiosciensce) against the murine I-A^d^ (clone 39-10-8), I-E^d^ (clone14-4-4S), and MHC class I (H-2^d^) (clone 34-1-2S) molecules were added to cultures and their capacity to inhibit peptide-specific proliferation were measured. Briefly, purified CD4^+^ T cells from mice primed with rLpp20 were preincubated with mAbs for 2 h at 37°C and then peptides(1.25 μg/ml) were added and incubated at 37°C, 5% CO2 for 5 days. The proliferation induced by peptides was tested as described above. Culture incubated with peptides without mAb served as negative control.

### Cytokine profile analysis by ELISA and real-time PCR

To detect the subset of CD4^+^ T cells, cytokine profile in response to peptide L1 and L2 was analyzed. CD4^+^ T cell culture supernatant (100 μl each) was collected after 72 h and cytokines were quantified by IFN-γ and IL-4 ELISA kits (eBioscience, San Diego, CA, USA). Cytokine production was calculated from the titration of supplied calibrated cytokines standards. Results were corrected for dilution of the sample to yield concentration in pg/ml. Meanwhile, mRNA expression level of IFN-γ and IL-4 were quantified. Total RNA from splenic lymphocytes was isolated using Trizol RNA isolation kit (Roche). Real time PCR was performed for IFN-γ, IL-4 and β-actin (as internal control) using SYBR Green Supermix in a 96-well plate of the ABI Prism 7500 Fast Sequence Detector (Applied Biosystems). 25 μl reaction mixtures contained 1 μl 100 X diluted cDNA, 12.5 μl 2X Power SYBR Master Mix (Applied Biosystems) and 150 nM of each primer. The primer sequences were listed as below:

IFN-γ: 5′-TGTCATCCTGCTCTTCTTTCTC-3′

5′-GACCTCAAACTTGGCAATACTC-3′

IL-4: 5′-AACTCAAGTGGCATAGATGTGG-3′

5′-GACCTCAAACTTGGCAATACTC-3′

β-actin: 5′-ATCCGTAAAGACCTCTATGCCAACA-3′

5′-GTCGCCTTCACCGTTCCAGTTT-3′

The relative fold change of mRNA level of IFN-γ and IL-4 was calculated using the delta delta Ct (threshold cycle) method [7]. The averages and standard deviations were determined from triplicate datasets. ΔCt is the difference between Ct of target mRNA and Ct of β-actin for each group.

### Flow cytometry analysis

Splenic lymphocytes stimulated with L1 and L2 respectively were phenotyped by double staining with anti-CD4 and CD3 mAbs (eBiosciensce) and fluorescence activated cell sorter. One million cells were collected and preincubated with rat IgG2b anti-mouse CD16/CD32 (clone 93, eBiosciensce) for 10 min to exclude unspecific Fc-receptor-mediated binding. After washing in cold PBS (2% bovine serum albumin), the cells were stained with PE-labeled anti-CD4 (clone RM4-5) and FITC-labeled anti-CD3 (clone 145-2C11) mAbs for 30 min on ice. Unspecific staining was controlled with appropriate FITC and PE labeled isotype controls respectively. The samples were analyzed with FACS Calibur flow cytometer and the data were analyzed by CELLQuest software (BD Biosciences).

### Statistical analysis

Data were representative of three independent experiments and expressed as mean ± standard deviation (SD). The results were processed using Student’s t-test with SPSS13.0 program. Significance was defined by a value of P < 0.05.

## Results

### Eight Th cell epitopes on Lpp20 were synthesized

To study Th epitopes on Lpp20, the potential binding motifs for I-A^d^ and I-E^d^ in amino acid sequence of Lpp20 were predicted respectively. Five I-A^d^ restricted and five I-E^d^ restricted epitopes were obtained (Table 1). Since there was only one amino acid different between residues 8 ~ 22aa and 7 ~ 21aa, the former was selected as candidate peptide because of its higher score. Residues 58 ~ 72aa was both I⇀A^d^ and I⇀E^d^ restricted epitope. Thus eight peptides were synthesized and named as L1 ~ L8. The purities of these peptides were all >90% except for L4 which was too hydrophobic to be measured (Table 1).

**Table 1:** **Prediction and synthesis of potential CD4**^**+**^**T cell epitopes on Lpp20**

**Name**	**Residues**	**Sequence**	**MHC restriction**	**Scores**	**Theoretical Mr**	**Actual Mr**	**Purity (100%)**
L1	83 ~ 97aa	**N**QA**T**A**K**ARANLAANL	I⇀A^d^	32	1526.72	1526.7	93.5
L2	58 ~ 72aa	**Y**EK**Y**S**G**VFLGRAEDL	I⇀A^d^	30	1746.94	1746.9	93.0
L3	12 ~ 26aa	**S**VI**A**A**M**VIVGCSHAP	I⇀A^d^	29	1454.77	1454.8	86.3
L4	8 ~ 22aa	**I**LG**M**S**V**IAAMVIVGC	I⇀A^d^	26	1476.91	1476.9	Crude
	7 ~ 21aa	**K**IL**G**M**S**VIAAMVIVG	I⇀A^d^	24			
L5	135 ~ 149aa	**K**EL**I**A**S**KM**L**ARYVGK	I⇀E^d^	22	1707.11	1707.1	95.6
L6	44 ~ 58aa	**A**PDV**V**GD**L**EKVAKY	I⇀E^d^	20	1689.93	1689.9	91.4
	58 ~ 72aa	**Y**EK**Y**S**G**VF**L**GRAEDL	I⇀E^d^	20			
L7	150 ~ 164aa	**D**RV**F**V**L**VG**L**DKQIVD	I⇀E^d^	20	1716.01	1716.0	90.7
L8	77 ~ 92aa	**D**VD**Y**S**T**NQ**A**TAKARA	I⇀E^d^	18	1610.78	1610.7	91.8

### L1 and L2 epitopes stimulated CD4^+^ T cell proliferation

**Figure 1:**
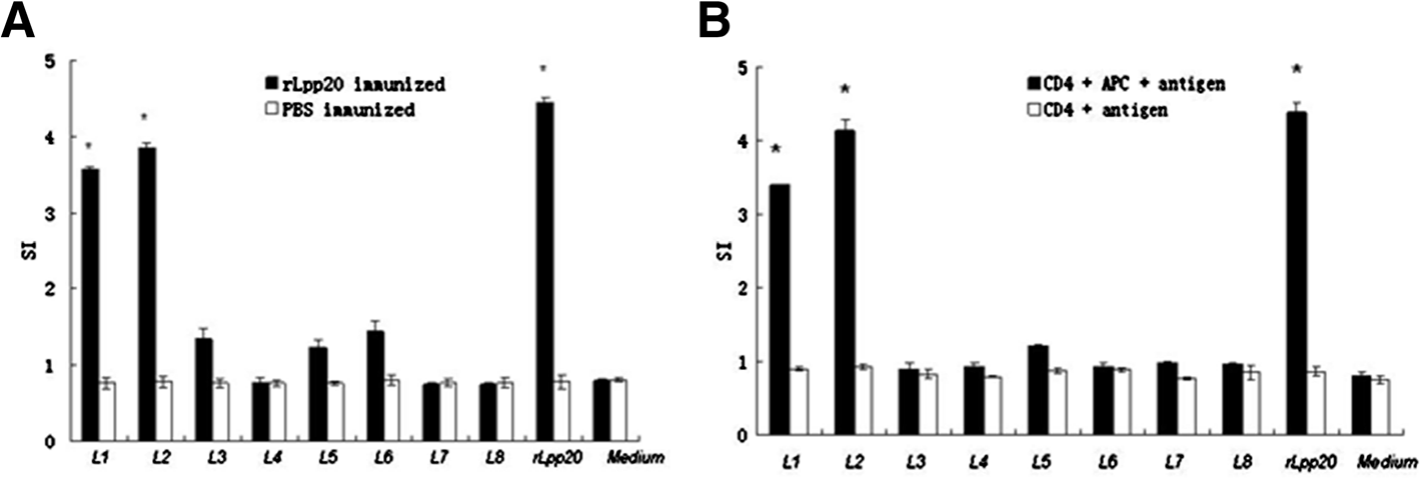
**Specific CD4**^**+**^**T cell proliferation analysis revealed that L1 and L2 were epitopes of Lpp20.** CD4^+^ T cells primed with rLpp20 were tested in proliferative responses to synthetic peptides (1.25 μg/ml), rLpp20 (15 μ g/ml) and medium as control. CD4^+^ T cells isolated from mice treated with PBS served as controls to determine if the responses were rLpp20-specific. Responses were expressed as mean Stimulation Index (SI) of three independent experiments ± S.D. SI ≥ 2 was considered as positive. (**A**) CD4^+^ T cell only responded to peptide L1, L2 and rLpp20 in presence of APC. (**B**) CD4^+^ T cell did not response to peptides and rLpp20 in absence of APC. (*) SI is higher than 2 at the 95% confidence level.

To identify Th cell epitopes on Lpp20, CD4^+^ T cells from mice immunized with rLpp20 were isolated and cultured with APCs and synthetic peptides. CD4^+^ T cells, which were isolated from rLpp20-immunized mice but not from PBS-treated mice, responded to L1, L2 and rLpp20 (SI > 2, Figure 1A). CD4^+^ T cells without APCs could not proliferate (SI < 2, Figure 1B). Thus, proliferative CD4^+^ T cell response was antigen-specific since the cells were primed by rLpp20 but not by PBS. Furthermore, CD4^+^ T cells recognize antigenic epitopes presented by APCs. These results indicate that L1 and L2 contain Th cell epitopes of Lpp20.

### CD4^+^ T cells recognized L1 and L2 epitopes in the context of MHC class II molecule

**Figure 2:**
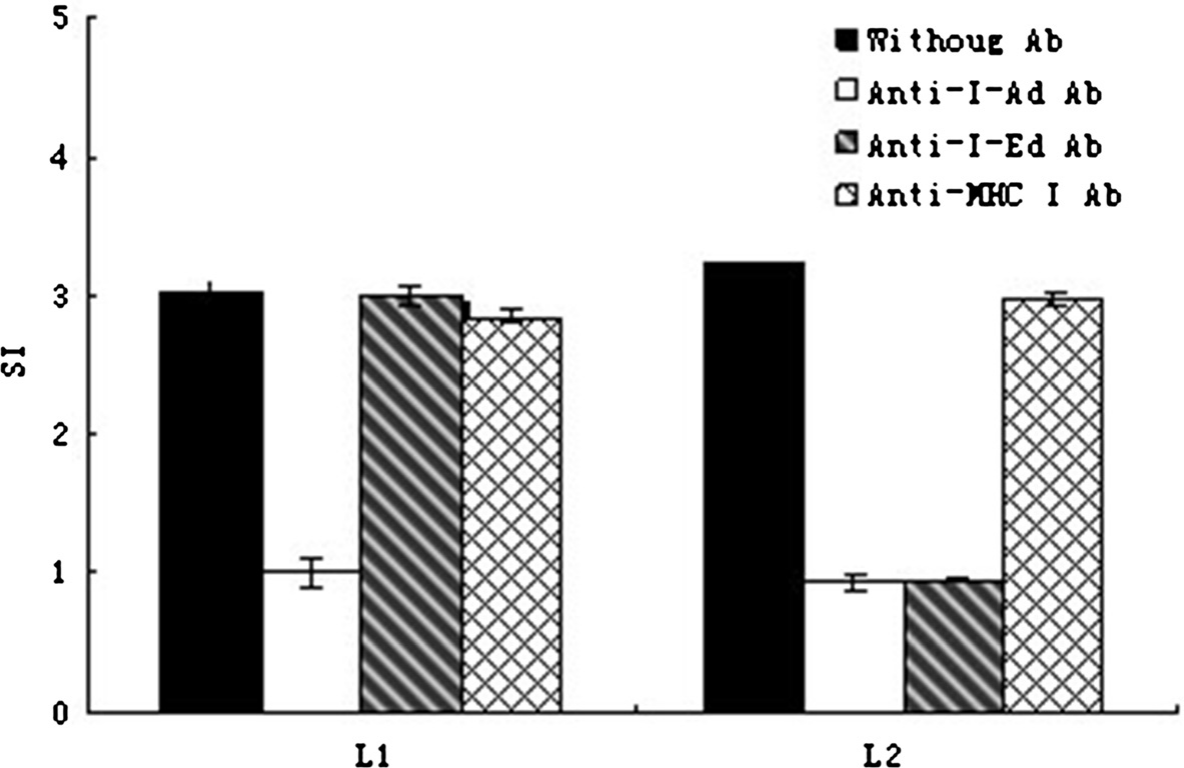
**Inhibition of Th cells proliferation by MHCII-specific mAbs.** Purified CD4^+^ T cells from mice primed with rLpp20 were stimulated with peptides (1.25 μg/ml) in the presence of mAbs specific for I-A^d^, I-E^d^, or MHCImolecules. Cultures incubated with peptides without mAbs were used as controls. The proliferation of the cells was quantified in triplicate by ^3^H-TdR incorporation. The values shown were means ± SD of three independent experiments.

CD4^+^ T cells recognize peptides presented by MHC II molecules on APCs. The interaction between CD4^+^ T cells and the peptide-MHC II complex can be blocked by mAbs against MHC II molecules. To determine the specificity of restriction of L1 and L2, anti-MHC mAbs namely I-A^d^ and I-E^d^ were incubated with APCs respectively prior to antibody stimulation of Th cells. Pretreatment of I-A^d^ specific mAb reduced cell proliferation significantly after L1 and L2 peptides’ stimulation. Meanwhile, I-E^d^ specific mAb only inhibited Th cells proliferation after L2 peptide stimulation but not L1 peptide. These results suggested that CD4^+^ T cells recognized L1 and L2 epitopes in the context of MHC II molecules and confirmed that L1 belonged to I- A^d^ restriction and L2 belonged to both I-A^d^ and I-E^d^ restriction. On contrast, anti-MHC I mAbs had no effect on L1 and L2 stimulated Th cells proliferation (Figure 2).

### L1 and L2 epitopes both mainly secreted Th1 cytokines

**Figure 3:**
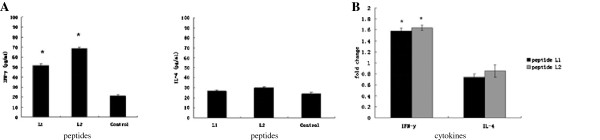
**Cytokine profile of splenic lymphocytes in response to peptide L1 and L2.** (**A**) CD4^+^ T cells from mice immunized with rLpp20 were incubated with each peptide for 72 h and cytokines in the culture supernatants were measured by sandwich ELISA. Medium without any antigen was used as control. Results were expressed as means ± S.D. of triplicate wells from three independent experiments. Significant difference from control was indicated by *(p < 0.05). (**B**) CD4^+^ T cells from mice immunized with peptide L1 and L2 was isolated. Relative levels of IFN-γ and IL-4 were determined by real-time PCR and fold changes calculated using the delta delta Ct method. CD4+ T cells isolated from mice treated with PBS in IFA were served as negative controls. The values shown were means ± SD of three experiments. Significant difference from PBS control group was indicated by * (p < 0.05).

To determine the subset of CD4^+^ T cells induced by L1 and L2 epitopes, cytokine profiles of CD4^+^ T cells in response to L1 and L2 were analyzed by ELISA. CD4^+^ T cells isolated from mice immunized with rLpp20 secreted two folds more Th1 specific cytokine (e.g. IFN-γ) after stimulation with L1 and L2 peptides compared with the control. Moreover, the level of the secreted Th2 specific cytokine IL-4 remained unchanged in the medium after L1 and L2 stimulation, suggesting Th1 was activated (Figure 3A).This observation was further confirmed in the splenic lymphocytes from L1 and L2 immunized mice. The mRNA level of IFN-γ was 1.5 fold change higher in the splenic lymphocytes stimulation than controls while the mRNA level of IL-4 remained unchanged upon L1 and L2 activation (Figure 3B). Therefore these results demonstrated that L1 and L2 preferentially elicited a polarized Th1-type response.

### The identified two peptides interacted additively

**Figure 4:**
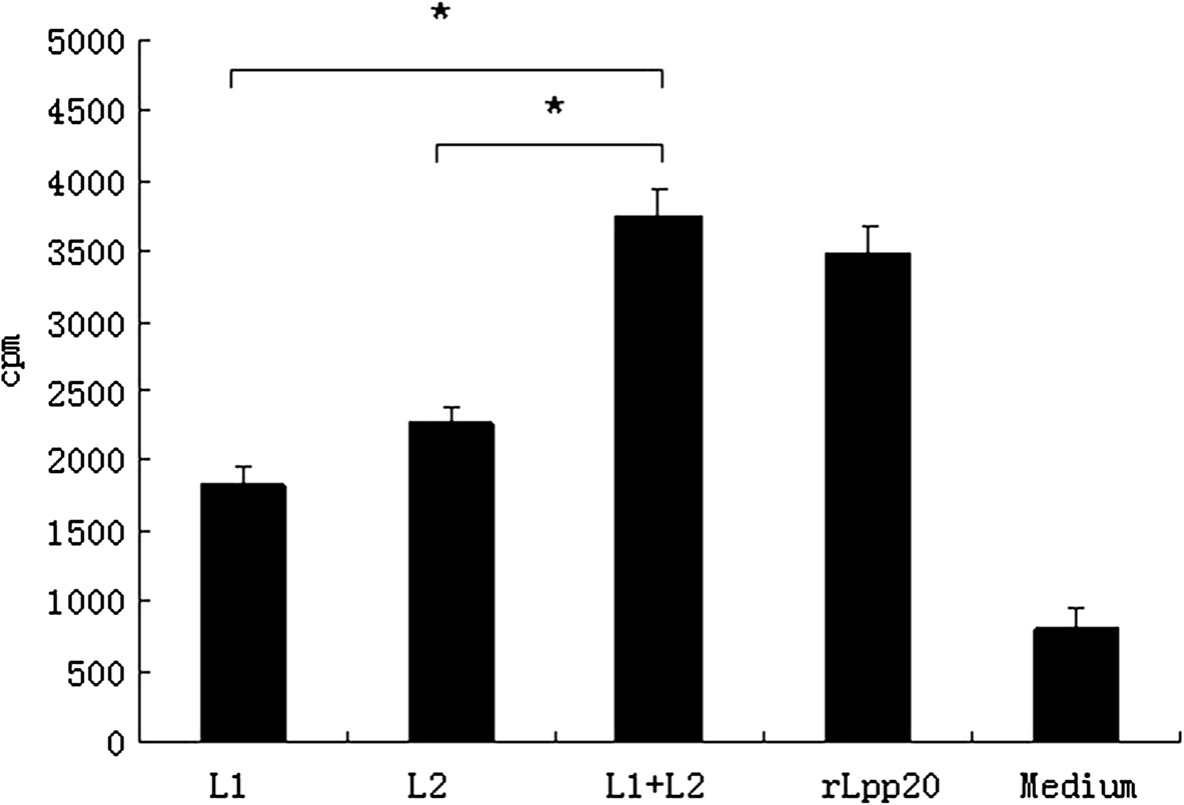
**Synergistic interaction of CD4+ T cell proliferation between two epitopes.** CD4+ T cells primed with rLpp20 were tested in a proliferation assay for responses to peptide L1, L2, pooled L1 and L2 (1.25 μg/ml) and rLpp20 (15 μ g/ml). Response to the antigen was expressed as the mean SI of three independent experiments ± S.D. The medium with rLpp20 was served as positive control and without any antigen weas used as negative control. The pooled peptides was significantly different from individual peptide (*p < 0.05). The values shown were means ± SD of three experiments.

To investigate if there was any additive or subtractive interaction between L1 and L2 epitopes, we pooled the two peptides to stimulate CD4^+^ T cells and compared the responses to the pooled peptides versus individual peptide. The pooled peptides stimulated significantly more elevated T cell response than each peptide (P < 0.05, Figure 4). Thus there is an additive interaction between the two peptides.

### CD4^+^ T cell from mice immunized with identified two epitopes exhibited proliferation

**Figure 5:**
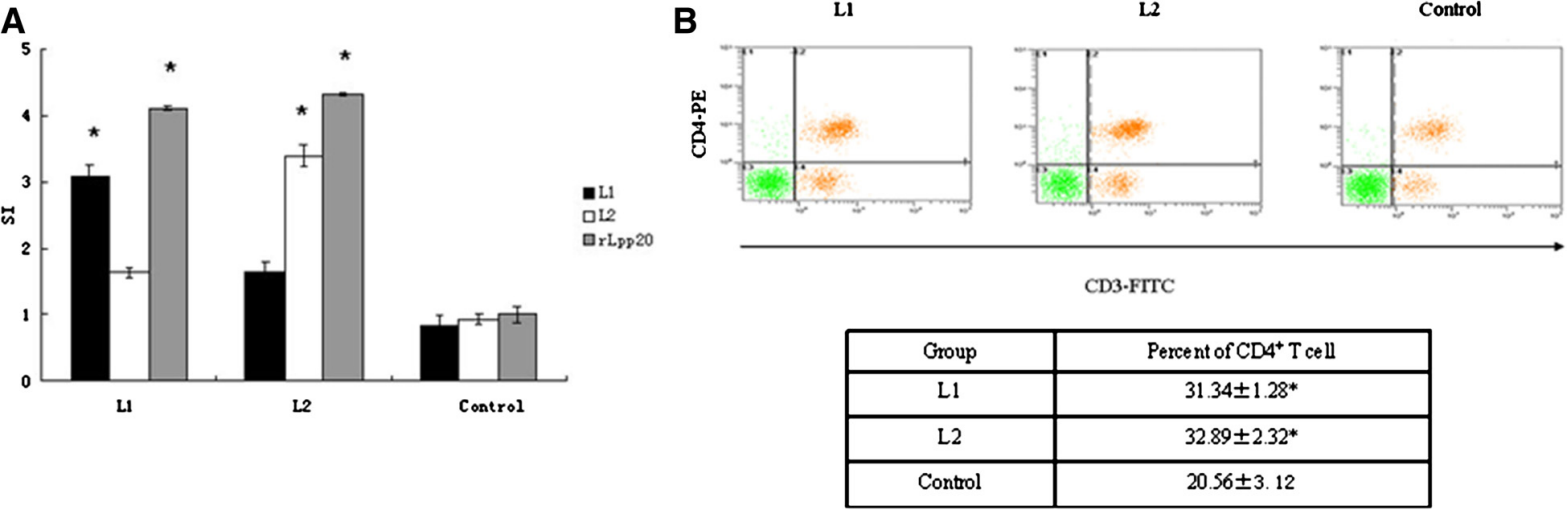
**Specific CD4**^**+**^**T cell responses in mice immunized with peptide L1 and L2.** (**A**) CD4^+^ T cells immunized with Lpp20-derived peptides were tested in proliferative responses to peptide L1 and L2 (1.25 μg/ml), rLpp20 (15 μg/ml) and control medium. CD4+ T cells isolated from mice treated with PBS in IFA were served as negative controls. Response to the antigen was expressed as mean SI of three independent experiments ± S.D. (*) SI was higher than 2 at the 95% confidence level. SI ≥ 2 was considered as positive. (**B**) FACS analyses were performed to determine the relative percentage of CD4^+^ CD3^+^ T cells immunized with peptide L1 and L2 respectively. Control group was immunized with PBS emulsified in IFA. All data were reported as means ± SD of three experiments. *p < 0.05 vs. control.

Having identified two Th cell epitopes on Lpp20, we then evaluated whether lymphocytes primed by L1 and L2 could recognize naturally processed antigen. For this purpose, we immunized mice with two peptides emulsified in IFA respectively and assessed the response of CD4^+^ T cells in vitro. T cells from mice immunized with L1 or L2 exhibited significant proliferation upon stimulation with L1, L2 and rLpp20 respectively in vitro (SI > 2, Figure 5). Under the same conditions, T cells from IFA without peptides-immunized mice did not respond to any peptide and rLpp20 (SI < 2, Figure 5A). This result suggested that Th1 cells induced by either L1 or L2 had responded to native antigen rLpp20. The FACS analyses were also performed to determine the relative percentage of CD4^+^ CD3^+^ T cells immunized with L1 and L2 respectively. The percentage of CD4^+^ CD3^+^ T cells from mice immunized with L1 and L2 were higher than control (P < 0.05, Figure 5B). These results confirmed again that L1 and L2 stimulated CD4^+^ T cells proliferation.

## Discussion

CD4^+^ T cells recognize antigenic epitopes in the context of MHC II molecules on APCs. Many investigations of *H.pylori* infection have demonstrated that the protective immune response is mediated by CD4^+^ T cells but not by CD8^+^ T cells. Therapeutic immunization reduced *H.pylori* colonization in stomach in mice lacking B cells, suggesting that T cell is protective [10]. Oral immunization with *H. pylori* whole-cell lysates reduced infection in wild-type and MHC I ^-/-^ mice, but it had no effect on MHC II ^-/-^ mice. Anti-*H.pylori* antibody levels in serum showed a dominant IgG in immunized wild-type and MHC I ^-/-^ mice but no detectable IgG in MHCII^-/-^ mice [1]. CD4^+^T cells from *H.pylori* antigen immunized mice were sufficient to transfer protective immunity to the immunodeficient recipients [11]. Taken together, identification and characterization of the Th cell eptioptes of Lpp20 would contribute to a better understanding of protective immunity to *H.pylori* and facilitate the development of effective immunotherapeutic and immunoprophylactic strategies.

The identification of CD4^+^ T epitiopes has traditionally been done either by sequencing of eluted peptides bound to specific MHC II molecules from APCs or by screening panels of overlapping peptides. These two methods were successfully performed to identify many T epitopes; however, the method of sequencing eluted peptide is potentially cumbersome to identify epitopes from multiple processed antigens but not specifically a single antigen. The overlapping peptide method needs synthesis of a series of overlapping peptides, thus making it an expensive, laborious and time-consuming process. In addition, this method possibly misses junctional epitopes that might be present in the overlapping regions, though spanning the entire length of the antigens [12]. These considerations lead us to use the combination of prediction and experiments to identify Th cell epitopes. Although it is possible to miss potential epitopes, this method represents a quick and effective approach to identify epitopes. Here we used algorithm program with T cell biological analysis to identify Th cell epitopes on Lpp20. Eight epitopes were selected to be synthesized and measured, of which two (L1 and L2) were identified to be Th cell epitopes, which were located at residues 83-97aa (L1) and 58-72aa (L2). Interestingly, L2, which was predicted to be both restricted by I-A^d^ and I-E^d^, effectively stimulated more proliferation of splenic CD4^+^ T cells than L1, which was predicted to be I-A^d^ restriction. Our results indicate that the combination of prediction and experiments is an easy and effective way to identify Th cell epitopes. The present findings may be valuable for the development of epitope-based vaccines against *H.pylori*.

CD4^+^ T cells can polarize to Th1 or Th2 cells based on their profile of cytokine. Th1 cells produce IFN-γ, IL-2 and TNF-β that mediate cellular immunity. In contrast, Th2 cells produce IL-4, IL-5, IL-10 and IL-13, which are responsible for humoral immunity [13]. It is generally believed that polarized Th1-type response is involved in the pathogenesis of *H.pylori* infection [14-17]. But Whether Th1 or Th2 type immune response is responsible for protective immunity is still unclear. Some studies support that Th2 response characterized by IL-4 secretion is important to clear *H.pylori*[18-21]. On the contrary, some studies reveal that protection against *H.pylori* is mediated by predominantly Th1-type immune responses independent of IL-4 [22,23]. Moreover, some researchers demonstrate that a mixed Th1-Th2 phenotype is correlated with the protective immunity against *H.pylori* infection [24]. Therefore, identification of Th1 and Th2 type epitopes may help us investigate the role of Th1 or Th2 type responses on pathogenesis and immunity of *H.pylori* infection. Meanwhile, it is also a pivotal step for a rational modulation of immune response by developing effective multiple Th1 or/and Th2 epitope vaccine. In this study, we found that L1 and L2 were both Th1-type epitopes and induced CD4^+^ T cell to mainly secrete IFN-γ.

In conclusion, we have identified two Th1 cell epitopes on Lpp20. Peptide L1 is I-A^d^ restricted epitope and peptide L2 is both I-A^d^ and I-E^d^ restricted epitopes. These two peptides both evoked Th1-type response. In addition, the two pooled peptides stimulated significantly more elevated T cell responses, showing an additive effect. We have identified one B cell epitope of Lpp20 using phage displayed library previously [6]. Next, we will combine these two Th epitopes and one B cell epitope to evaluate whether the combined epitopes can induce protective response and prevent *H.pylori* infection better than the whole Lpp20 protein in BALB/c mice. The data from our mouse model will provide significant information for further study of epitope-based vaccine.

## Conclusions

We identified two H-2^d^ restricted CD4^+^ T cell epitopes of *H.pylori* Lpp20, which interacted additively and mainly secreted INF-γ and belonged to Th1 type epitopes. Immunization with the identified epitopes primed antigen-specific CD4^+^ cell response. These data provide useful insights regarding immunity against *H.pylori* and have the potential to guide the design of epitope-based vaccines.

## Competing interests

The authors declare that they have no competing interests.

## Authors’ contributions

YL designed and contributed the majority of the bench work. YJ, YX and SZ expressed the recombinant Lpp20 and participated in real-time PCR and ELISA. LZ and SZ analyzed the statistical data for the experiments. JL immunized the animals. DH isolated and culture CD4^+^ T cell. YN designed the experiments. YL wrote the manuscript and all authors have read and approved the final manuscript.
